# Internal fistula stenosis with true pseudoaneurysm formation in a patient on maintenance hemodialysis: A case report

**DOI:** 10.1097/MD.0000000000038111

**Published:** 2024-05-10

**Authors:** Xiaoya Tong, Yan Ran, Jingjing Da, Ying Hu, Jing Yuan, Yan Rui

**Affiliations:** a College of Clinical Medicine, Guizhou Medical University, Guiyang, China; b Department of Nephrology, People’s Hospital of Guizhou Province, Guiyang, China; c Guizhou Provincial Institute of Nephritic and Urinary Disease, Guiyang, Guizhou, China; d Department of Nephrology, The Affiliated Hospital of Guizhou Medical University, Guiyang, China.

**Keywords:** arteriovenous fistula, hemodialysis, open surgery, pseudo aneurysm, true aneurysm

## Abstract

**Background:**

Arteriovenous fistula stenosis can directly lead to the formation of autologous arteriovenous fistula aneurysms (AVFAs), but the coexistence of true and pseudoaneurysms is relatively rare. The coexistence of true and pseudoaneurysms increases the risk of rupture of the arteriovenous fistula and complicates subsequent surgical intervention, potentially posing a threat to the patient’s life, and thus requires significant attention.

**Case presentation:**

The patient presented with arteriovenous fistula (AVF) after hemodialysis 6 years ago. 2 years ago, the patient presented with a mass that had formed near the left forearm arteriovenous fistula and gradually increased in size. Preoperatively, the AVF stenosis was identified as the cause of the mass formation, and the patient was operated on. First, the blood flow was controlled to reduce the pressure at the aneurysm, and then the incision was enlarged to separate the AVF anastomosis from the mass area. The stenotic segment of the true and pseudo aneurysms and cephalic vein was removed and the over-dilated proximal cephalic vein was locally narrowed and subsequently anastomosed with the proximal radial artery to create AVF. The patient was dialyzed with an internal fistula the next day and showed no clinical manifestations related to end-limb ischemia.

**Conclusion:**

We removed a true pseudoaneurysm in AVF and secured the patient’s vascular access. This report provides an effective strategy to manage this condition.

## 1. Introduction

Arteriovenous fistula (AVF) is now used as the first choice when constructing vascular access because of its low infection rate and low thrombosis rate.^[1]^ However, only 50% of AVFs remain functional 6 months after creation,^[2]^ and the main causes of their dysfunction are vascular stenosis and thrombosis.^[3]^ Arteriovenous fistula stenosis can lead directly to the formation of autologous arteriovenous fistula aneurysms (AVFAs). Partial aneurysm resection and repair reconstruct vascular access by removing unhealthy or excessive tissue and using the original vessel wall as much as possible,^[4]^ which has the advantage of eliminating the need for transition to a dialysis catheter and fewer complications.^[5]^ However, clinical work with stenosis leading to the coexistence of true and pseudo aneurysms is rare. We summarize the diagnosis and management of a patient with confirmed endovascular stenosis forming both true and pseudoaneurysms.

## 2. Case presentation

**Figure 1.**
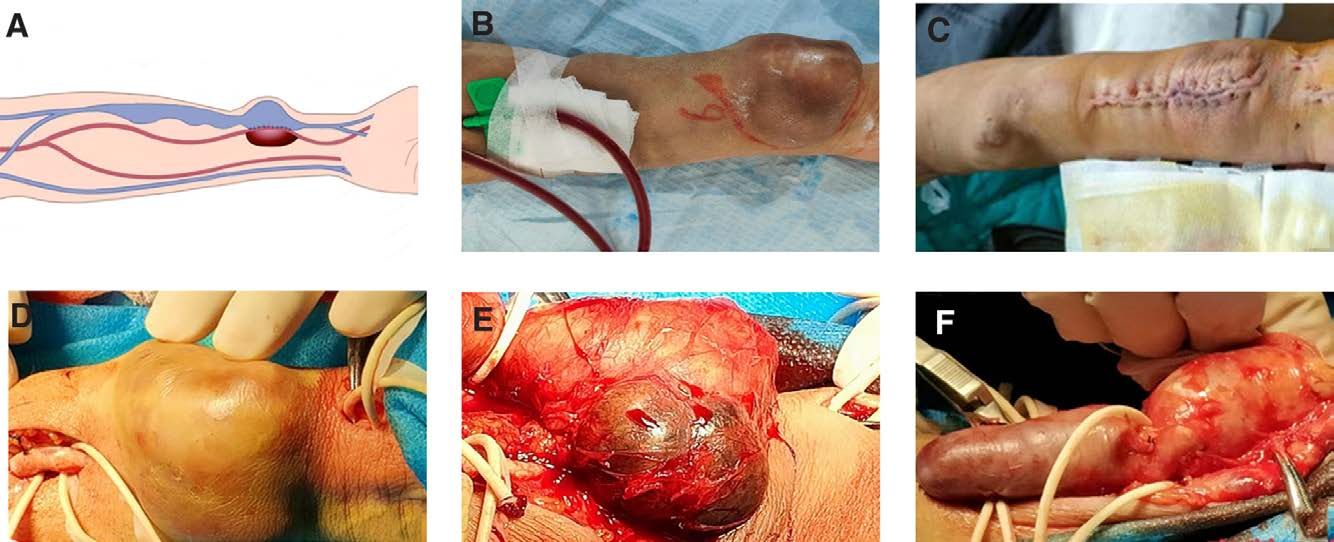
Simplified diagram of the hemodialysis access in the present case (A). Appearance of the left forearm before and after surgery (B and C). Free out of upstream and downstream arteries (D). Both true and pseudo aneurysm (E). Venous stenosis (F).

A 40-year-old female patient was admitted to the hospital in October 2021 with localized pain from an arteriovenous fistula. 2015, the patient was clearly diagnosed with end-stage renal disease due to glomerulonephritis, and in the same year, she underwent left forearm arteriovenous fistuloplasty and regular hemodialysis. Since 2019, the patient presented with no obvious cause for the formation and gradual increase in size of a mass near the left forearm arteriovenous endovascular fistula with pulsating sensation and local pain, which was tolerable. There was also high venous pressure during dialysis and difficulty in hemostasis after the patient being punctured, which suggested that the vein was chronically under high pressure. The patient was not further treated during this period because of poor compliance. Physical examination revealed a hemangioma-like mass formation at the endovascular fistula of the left forearm, about 5 × 6 cm in size (Fig. 1A and B), with palpable tremor and pulsation, and audible vascular murmur. After admission, the patient underwent surgical treatment, resulting in the removal of the mass while preserving the functionality of the arteriovenous fistula in the left forearm (Fig. 1C). The following describes the detailed surgical procedure. The result of Allen test of the patient’s left upper extremity was negative and the patient was treated with symptomatic support before the surgery. In combination with the patient’s physical examination and ultrasound findings, the AVF vein stenosis was considered to be the cause of the mass formation, and the distance between the stenosis and the fistula was about 3 cm. After local anesthesia and routine disinfection of the surgical area, 2 longitudinal incisions, each approximately 8 to 10 mm, were made approximately 1 cm distal to the AVF and approximately 3 cm proximal to the AVF. The distal end of the internal fistula and the proximal radial artery were separated (Fig. 1D), and the artery was ligated to control blood flow to reduce the pressure at the aneurysm, and then the incision was enlarged to approximately 7 cm to separate the AVF anastomosis and the area of the mass, and the coexistence of true and pseudo aneurysms was seen intraoperatively (Fig. 1E).

**Figure 2.**
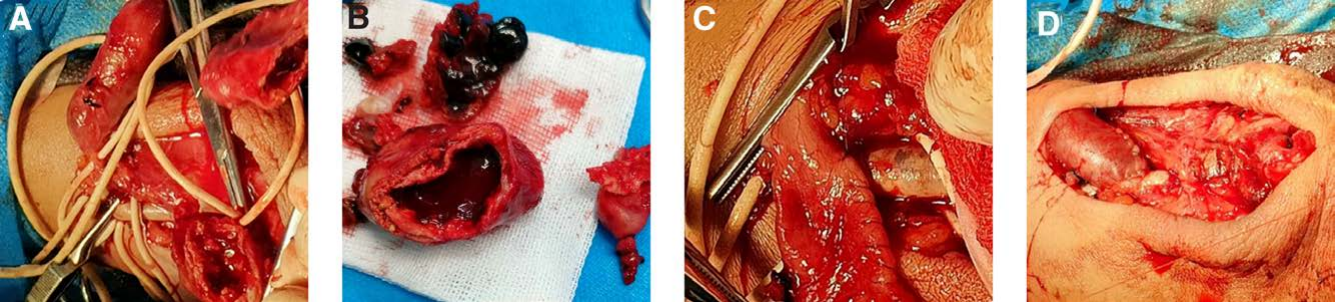
Removal of true and pseudo aneurysm (A and B). Narrowing of over-dilated veins (C). reestablishing AVF (D). AVF = arteriovenous fistula.

Intraoperatively, the stenotic segment of the cephalic vein was separated (Fig. 1F), and the true pseudoaneurysm and the stenotic segment of the cephalic vein were excised (Fig. 2A and B). To prevent possible ischemia at the end of the limb due to excessive blood flow in the dilated cephalic vein, the proximal dilated cephalic vein was locally narrowed by applying 6-0 Prolene (Fig. 2C), which was approximately 1 cm in length, and later anastomosed with the proximal radial artery (Fig. 2D). Postoperative AVF venous tremor was evident, and the patient was dialyzed with an internal fistula the next day without clinical manifestations related to end-limb ischemia. The patient was followed up 1 month after the operation, and dialysis was smooth and blood flow could meet the dialysis requirements.

## 3. Discussion

The first choice of vascular access for maintenance hemodialysis patients is an autologous AVF.^[6]^ Thrombosis and stenosis are the main causes of access failure. Early detection of access stenosis and timely percutaneous transluminal angioplasty and/or open surgical intervention may prolong the life of the vascular access.

Arteriovenous endovascular fistulas are classified into 4 types according to the type of clinical stenosis: type I for stenosis within 5 cm of the anastomosis or anastomosis; type II for stenosis at the dialysis needle puncture, stenosis between puncture points, or stenosis at multiple longer punctures; type III for stenosis at the vascular confluence; and type IV for stenosis at the artery.^[7]^ Open surgical treatment is more effective mainly for type I stenosis. Since 2002, the number of patients opting for percutaneous transluminal angioplasty treatment at AVF stenosis far exceeds that of open surgery.^[8]^ However, balloon dilation leads to vascular injury and vessel wall thickening, which predisposes to the development of restenosis or recurrent stenosis or even the need for repeated interventions,^[9]^ and the restenosis rate is 2 to 2.5 times higher than that of open surgery.^[10]^

In this patient’s case, we can see that the patient’s venous stenosis is within 5cm of the anastomosis, which is type I stenosis, and the patient has a good surgical result. This patient has a combined stenosis inferior vasodilatation, forming a true aneurysm of the autogenous AVFAs. The relevant expert consensus defines AVFAs as dilatation occurring months or years after surgery, with pulsation, a tumor wall containing the entirety of the vessel wall, and a tumor internal diameter > 3 times or more the adjacent normal vessel internal diameter and an internal diameter > 2 cm,^[11,12]^ and the diagnosis of this patient was clear according to the expert consensus.

AVFAs are not currently typed by guidelines or expert consensus. The available literature reports the Valenti typing and Balaz typing. Valenti staging divides AVFAs into 4 types: type I: no hump-like AVFAs, type Ia: veins mostly uniformly dilated throughout the anastomosis; type Ib: dilated within 5 cm of the proximal end of the anastomosis. Type II: hump-like aneurysm, type IIa: at least 1 localized dilatation of the vein, commonly 2, in a classic hump-like dilatation, mostly associated with the dialysis puncture site, with a normal or narrow diameter vein between the aneurysms; type IIb: both postanastomotic aneurysm and localized dilatation of the puncture, a combination of Type Ib and Type IIa. Type III: complex or specific type of aneurysm without typical features. Type IV: pseudoaneurysm. In Valenti staging, both type I and type IV were present in this patient.

Balaz staging is based on stenosis or thrombosis detected by ultrasound or contrast. Balaz Type I: no stenosis or thrombosis; type II: stenosis of > 50% of vessels, which can be classified as inflow artery stenosis (IIa), anastomotic stenosis (IIb), outflow tract stenosis (IIc) or central venous stenosis (IId); type III: >50% of vessels with thrombosis; type IV: total lumen thrombosis.^[13]^ This patient has only stenosis but no intravascular thrombosis, so the type belongs to Balaz typing type II.

It was found that different treatment modalities should be used depending on the location, size, and cause of AVFAs. Limited AVFAs occurring at the anastomotic site can be treated by resection of the aneurysm and AVF reconstruction proximal to the anastomosis, which does not reduce the number of AVF puncture sites and is available postoperatively, avoiding the need for central venous puncture placement. AVFAs occurring at the proximal end of the anastomosis or at the puncture site can be treated with partial resection of the AVFAs wall + reduction angioplasty.

Partial aneurysm resection and repair is a targeted surgical approach to reconstruct vascular access by removing unhealthy or excessive tissue and using the original vessel wall as much as possible, with the advantage of being simple, effective and maintaining AVF patency without the use of a graft vessel, without the need for transition to a dialysis catheter, and with low complications.^[14]^Removal of the thin skin and subcutaneous tissue involved on the surface of the aneurysm not only improves the appearance of the skin but also enhances the restriction of blood vessels, and tension in the surgical area and reduces the probability of poor postoperative incisional healing.^[14,15]^

AVFAs are a common complication of late AVF. The patient’s AVFAs did not increase rapidly, but there was a local rupture of the endovascular fistula due to stenosis and a pseudoaneurysm formation at the endovascular opening, which was at risk of local rupture. For limited AVFA occurring at the anastomosis, the aneurysm can be removed and AVF reconstruction performed proximal to the anastomosis; this procedure allows the patient to use vascular access for hemodialysis immediately postoperatively, avoiding the need for central venipuncture. This surgical treatment offers the advantages of simplicity, effectiveness, and preservation of AVF patents, and does not require the use of graft vessels, does not require the use of dialysis catheters for transition, and has low complications.

This case also highlights certain limitations in our practice, necessitating attention to patient health education and disease monitoring in our daily work. This is to encourage patients to seek medical attention as early as possible, preventing the coexistence of true and pseudoaneurysms, thus reducing the risk of arteriovenous fistula rupture and ensuring the safety of the patient’s dialysis access.

## 4. Conclusions

A 40-year-old female patient, who has been on regular dialysis for 6 years, underwent surgical treatment for the coexistence of true and pseudoaneurysms. Although the surgery alleviated related risks and preserved the functionality of the original arteriovenous fistula, we still need to focus on patient health education and disease monitoring in our daily work.

## Acknowledgment

We thank the Department of Nephrology, Guizhou Provincial People’s Hospital (Guiyang, China) for technical support of this study.

## Author contributions

**Data curation:** Yan Ran.

**Methodology:** Jingji Da, Ying Hu.

**Project administration:** Yan Rui.

**Resources:** Xiaoya Tong, Yan Ran, Jingji Da, Ying Hu, Jing Yuan.

**Writing – original draft:** Xiaoya Tong.

**Writing – review & editing:** Jing Yuan, Yan Rui.
